# Risk Factors for Positive Margins in Breast-Conserving Surgery

**DOI:** 10.7759/cureus.38399

**Published:** 2023-05-01

**Authors:** Hussain A Abdulla, Basma Rajab, Maryam Hammad, Amal Alrayes

**Affiliations:** 1 Surgery, Salmaniya Medical Complex, Manama, BHR; 2 Pathology, Salmaniya Medical Complex, Manama, BHR; 3 Surgical Oncology, Alkindi Hospital, Zinj, BHR

**Keywords:** wide local excision, lumpectomy, breast cancer, breast conserving surgery, positive margins

## Abstract

Introduction

Breast-conserving surgery (BCS) followed by adjuvant radiotherapy has similar overall survival compared to mastectomy but is associated with higher rates of local recurrence. Positive surgical margins in BCS are the most important predictor of local recurrence. The aim of our study was to assess the risk factors associated with positive margins in women undergoing BCS for breast cancer in order to inform our clinical practice and minimize re-operation rates.

Methods

Patients with a diagnosis of breast cancer who underwent BCS from January 2013 to January 2021 were identified from our pathology database and included in the study. All patients underwent a lumpectomy with the removal of additional shaved cavity margins. Statistical analysis was used to assess the effect of patient clinical and pathological risk factors on the rate of positive margins.

Results

One hundred and twenty patients underwent BCS for breast cancer. Twenty-four percent of patients had positive margins. Of the 29 patients that underwent subsequent re-excisions, only 13 (45%) had residual disease in the re-excision specimen. In younger patients, tumors localized in lower quadrants and the presence of extensive intraductal component within invasive breast cancer increased the risk of positive margins. In addition, positive margins were encountered more significantly in patients with ductal carcinoma in situ (DCIS) compared to invasive tumors. Multivariate analysis showed that DCIS and young age were the only factors independently associated with positive margins.

Conclusion

DCIS and younger patients have a higher rate of positive margins during BCS than invasive breast cancer. For such patients at higher risk of positive margins, excision of cavity shave margins and intraoperative inking may be done to lower positive margin rates. Preoperative review of breast imaging, core biopsies, and counseling of patients about the likelihood of positive margins is important.

## Introduction

For patients with early breast cancer, breast-conserving surgery (BCS) with adjuvant radiotherapy is considered the treatment of choice, with similar overall and disease-specific survival rates but a higher local recurrence rate compared to patients who undergo a mastectomy [1,2]. Having negative surgical margins is important in BCS because patients with positive margins have a greater risk of local recurrence and decreased disease-specific survival [3]. The guidelines set by the American Society of Clinical Oncology (ASCO), the American Society for Radiation Oncology (ASTRO), the Society of Surgical Oncology (SSO), and the National Comprehensive Cancer Network (NCCN) advocate adequate margins of no tumor on ink for cases of invasive carcinoma and 2 mm for patients with ductal carcinoma in situ (DCIS) [4,5]. Positive margins are encountered in 5-57% of patients undergoing BCS, and the rate of repeat surgery ranges from 10-40% [6]. Patients with positive margins require subsequent re-excision of margins, resulting in poor cosmetic outcome, patient anxiety, increased cost, complications, delay in starting adjuvant treatment, and even in mastectomy [7]. In addition, 30-65% of patients with R1 resection margins do not have residual disease upon re-excision [8].

Previous studies have reported predictors of local recurrence and the risk factors associated with positive margins in lumpectomy specimens [9-13]. However, these studies have a diverse study population with various surgical techniques and different guidelines for adequate resection margins [7]. For these reasons, the aim of our study was to determine risk factors associated with positive margins within our institution in order to inform our clinical practice of BCS and minimize re-operation rates.

## Materials and methods

Patients with invasive breast cancer and DCIS who underwent BCS at our institution between January 2013 and January 2021 were identified from our pathology database and included in the study. The study method was reviewed and approved by the Ministry of Health Research Ethics Committee in Bahrain. Patients who underwent diagnostic excisional biopsies and mastectomies and those who achieved complete pathological response after treatment with neoadjuvant systemic therapy were excluded. 

For patients, who met the inclusion criteria, histology reports were retrieved from our institution's electronic medical records, and the following data was retrospectively reviewed: age at diagnosis, laterality, quadrant, focality, histologic type, grade, tumor size, nodal status, evidence of extratumoral DCIS, lymphovascular invasion (LVI), the status of hormone receptors, human epidermal growth factor receptor 2 (HER2) expression, Ki67 proliferation index, history of neoadjuvant chemotherapy, wire localization and presence of microcalcifications.

All patients underwent a lumpectomy with the removal of additional shaved cavity margins. The extra margins were excised from the wide local excision cavity wall after the lumpectomy specimen was removed and sent for histology. Depending on the extent of wide local excision, these included additional posterior, anterior, lateral, medial, inferior, and superior cavity margins. The majority of patients had excision of all six extra margins. We oriented our lumpectomy specimens by silk sutures, with a short stitch for the superior margin, a long suture for the lateral margin, and double sutures for the deep margin (if anterior skin was not excised with the lumpectomy specimen). We used skin clips (placed on the tumor side) to mark every extra margin. For patients with non-palpable lesions, preoperative localization was done by stereotactic- or ultrasound-guided wire localization (depending on radiologist preference), usually on the morning of surgery. Radiological assessment of tumor margins was assessed by ultrasonography or mammogram for non-palpable lesions. If a close margin was identified, we proceeded immediately with further excision during surgery. Pathological assessment of tumor margins by frozen section was not performed intraoperatively.

Lumpectomy specimens were reviewed by consultant pathologists to confirm margin status. Margins were considered negative according to the current ASCO/SSO/ASTRO and NCCN guidelines of "no ink on tumor" for invasive cancer (with or without DCIS) and a margin of >2 mm for DCIS. Patients who had margins involved by atypical ductal hyperplasia, atypical lobular hyperplasia, or lobular carcinoma in situ were considered negative. 

Rates of positive margins among different patient groups were compared in terms of patient and tumor characteristics. We used Fisher's exact test or chi-square test to assess differences between clinicopathological risk factors and positive margins. Statistical analysis was performed by the SPSS package (IBM, Inc., Armonk, US). We considered a p-value of less than 0.05 as a significant value.

## Results

Baseline patient and tumor characteristics are summarised in Table 1. One hundred and twenty females with breast cancer were eligible for inclusion in the study. The mean age at diagnosis was 53 years. Breast cancer was most likely to occur in the upper outer quadrant (UOQ), with a frequency of 68%. Most of the patients (82%) had unifocal disease. Invasive ductal carcinoma was the predominant histological subtype (75%). Sixty-one percent of DCIS tumors and 31% of invasive tumors were reported to be high-grade. While only 15% of lumpectomy specimens were pure DCIS, there was the presence of an intraductal component in 59% of patients with invasive breast cancer. Axillary lymph node metastasis was detected in 28% of the patients. Only 22% of specimens showed the presence of lymphovascular invasion (LVI). Ninety-eight patients were found to have hormone-positive (either estrogen receptor [ER] or progesterone receptor [PR} receptor) breast cancer. Only 17% of patients with invasive breast cancer showed HER2 amplification. Almost a quarter (24%) of our patients had positive margins. Of the 29 patients that underwent subsequent re-excisions, only 13 (45%) had residual disease in the re-excision specimen.

**Table 1:** Baseline characteristics of the study population LIQ - lower inner quadrant; LOQ - lower outer quadrant; UIQ - ​​​​​​​upper inner quadrant; UOQ - ​​​​​​​upper outer quadrant; DCIS - ​​​​​​​ductal carcinoma in situ; IDC - ​​​​​​​invasive ductal carcinoma; ILC - ​​​​​​​invasive lobular carcinoma; N/A - ​​​​​​​not available; LVI - ​​​​​​​lymphovascular invasion; ER - ​​​​​​​estrogen receptor; PR - ​​​​​​​progesterone receptor; HER2 - ​​​​​​​human epidermal growth factor 2 receptor

Age	Number (n=120)
Mean	53
Median	52
Range	31-76
Tumor side	
Right breast	59
Left breast	61
Quadrant	
Central	10
LIQ	8
LOQ	6
UIQ	14
UOQ	82
Disease focality	
Unifocal	98
Multifocal	22
Tumor type	
DCIS	18
IDC	90
ILC	4
Mucinous carcinoma	3
Medullary carcinoma	2
Papillary carcinoma	1
Tubular carcinoma	1
Mixed IDC and ILC	1
DCIS grade	
Low	5
Intermediate	2
High	11
Invasive tumor grade	
Grade I	20
Grade II	50
Grade III	32
Extratumoral DCIS	
Present	60
Not present	42
N/A	18
T stage	
Tis	18
T1	47
T2	49
T3	6
Nodal status	
N0	87
N1	21
N2	9
N3	3
LVI	
Present	27
Not present	93
ER	
Positive	97
Negative	23
PR	
Positive	92
Negative	28
HER2	
Positive	28
Negative	80
N/A	12
Ki67 index	
≤20%	80
>20%	28
N/A	12
Neoadjuvant therapy	
Yes	11
No	109
Wire localization	
Yes	31
No	89
Microcalcifications	
Present	50
Not present	70
Margins	
Involved	29
Not involved	91

A comparison of clinical and pathological risk factors of the patients is shown in Table 2. Risk factors, such as tumor laterality, multifocality, histology, grade, tumor diameter, nodal status, LVI, ER, PR, Ki67 index, neoadjuvant therapy, wire localization, and microcalcifications did not show a statistically significant difference between patients with positive and negative margins. Although HER2 expression was associated with a slightly higher rate of positive margins, the difference was also not statistically significant. Nevertheless, in younger patients (p=0.018), disease localized to lower quadrants (p=0.015) and the presence of extensive intraductal component within invasive breast cancer (p=0.004) increased the probability of finding positive margins. In addition, positive margins were encountered in a significantly greater number of patients with DCIS (p=0.002) compared to invasive breast cancer. On multivariate analysis, DCIS and young age were the only factors independently associated with positive margins (p<0.05).

**Table 2:** Relationship between clinicopathological features and risk of positive margins DCIS - ductal carcinoma in situ; LVI - ​​​​​​​lymphovascular invasion; ER - ​​​​​​​estrogen receptor; PR - ​​​​​​​progesterone receptor; HER2 - ​​​​​​​human epidermal growth factor receptor 2

Age	Positive margins (%)	p-value
≤50	35%	0.018
>50	13%	
Tumor side		
Left	19.7%	0.289
Right	25.4%	
Quadrant		
Lower	43%	0.015
Upper	16%	
Multifocality		
Yes	14%	0.588
No	24%	
Histology		
Ductal	22%	0.610
Lobular	25%	
Tumor grade		
Low	25%	0.375
High	19%	
Tumor diameter		
≤20 mm	29%	0.524
>20 mm	16%	
Intraductal component		
Yes	18%	0.004
No	14%	
Tumor type		
DCIS	55%	0.002
Invasive	17%	
Nodal status		
Node-positive	21%	0.582
Node-negative	23%	
LVI		
Present	26%	0.495
Not present	22%	
ER		
Positive	22%	0.791
Negative	23%	
PR		
Positive	22%	0.906
Negative	25%	
HER2		
Positive	21%	0.053
Negative	19%	
Ki67 index		
≤20%	50%	0.255
>20%	16%	
Neoadjuvant therapy		
Yes	9%	0.732
No	24%	
Wire localization		
Yes	26%	0.811
No	21%	
Microcalcifications		
Present	22%	0.971
Not present	23%	

## Discussion

In our study, we found that patients who underwent BCS for pure DCIS had a significantly higher rate of margin positivity than those with invasive cancer. Patients who were younger or had tumors in the lower quadrants were more likely to have positive margins. In patients with invasive disease, those who had extensive intraductal component were also more likely to have positive margins. Previous studies have described several risk factors for positive margins in BCS. These include patients with DCIS, multifocal tumors, lobular histology, presence of extensive intraductal component, axillary lymph node metastasis, HER2 amplification, tumor size, lower quadrant tumors, and microcalcifications on mammography [11-13]. The positive margin rate in our study was 24% and is within the literature range of 5-57% [6].

Only 45% of patients that underwent repeat surgery had residual disease in the re-excision specimens. In one study, it was reported that the use of electrocautery at the index operation may destroy tumor cells at the resection margin, resulting in no residual tumor in the re-excision specimen [7]. Another study noted the inflammatory response at surgical wounds as another reason for the absence of residual cancer cells [14]. Nevertheless, performing completion mastectomy after initial BCS increases the probability of finding residual disease [7]. Younger women are more likely to have positive margins due to the reduced sensitivity of ultrasound and mammogram in these patients, which in turn, may not allow the surgeon to accurately define the disease extent prior to surgery [15]. The age of the patient may also influence the type of surgery, as there is a preference for BCS in younger women [16].

Low residual disease rates in the re-excision specimen can also be caused by techniques associated with specimen orientation [7]. Specimen orientation of the lumpectomy specimen is crucial for determining the site of the involved margin when planning for second surgery re-excise residual disease. In our institution, lumpectomy specimens are designated with a short superiorly placed stitch for the superior margin and a long stitch marked laterally to mark the lateral margin. Furthermore, two double sutures may be placed on the posterior aspect of the specimen in order to designate the deep margin if the skin is not removed. This suture technique may lead to confusion between the pathologist and surgeon regarding the orientation of the specimen, thereby resulting in the excision of the wrong surgical margin and leaving behind residual disease [15]. Intraoperative inking of all margins has been proposed to enhance specimen orientation to guide re-excisions for positive margins [17]. While the technique of specimen orientation has no effect on the rates of positive margins, intraoperative inking is considered superior to suture orientation for residual disease identification at re-excisions [15]. Therefore, proper orientation and inking of the lumpectomy specimen may allow for better positive margin identification, thereby restricting re-excision only to that margin [7].

Intraoperative frozen section, along with other techniques, including oncoplastic surgery, cavity shave margins, and wire-guided localization, may help to reduce positive margins in BCS [6]. Some centers use frozen section to assess margins intraoperatively. However, this technique has not been universally found to reduce positive margins, and concerns with the use of intraoperative frozen section include the possibility of false negative results, the need for experienced pathologists, increased operative time, and the inability to sample the entire lumpectomy specimen intraoperatively to provide a comprehensive margin assessment [18].

In our study, wire-guided localization was not associated with lower rates of positive margins. Radioactive seed localization has recently emerged as an alternative to wire localization for non-palpable tumors. Studies comparing wire localization to radioactive seed localization have conflicting results, with some authors reporting that radioactive seed localization is superior and others findings that both techniques are comparable in terms of positive margins and re-excision rates [6,19]. There has been consistent evidence from the literature that routine excision of cavity shave margins results in at least a 50% reduction in the positive margin rate [20]. In our experience, this technique takes less than 10 minutes to perform and is not associated with an increase in complications or worse cosmetic results.

Limitations of our study include its single surgeon experience, retrospective nature, relatively small sample size, and absence of other factors, such as breast size, the density of breast tissue, the surgical technique of other surgeons, genetic predisposition, and imaging review with Breast Imaging Reporting and Data System (BI-RADS) score to evaluate their influence on margin status. However, understanding risk factors for positive margins can help change our practice of BCS in order to lower positive margin rates and avoid a second operation because they can be assessed preoperatively by imaging and core biopsy [8]. Radiotherapy with a boost dose to the tumor bed for patients with involved margins may be an option, especially if the re-excision is technically not feasible or if the patient is unfit or at high risk for second surgery [7]. As part of informed consent, it is very important to explain to patients about likelihood of positive margins with BCS and the possible need for a second surgery. For patients at higher risk of positive margins, we suggest routine circumferential cavity shaving to help decrease positive margin rates.

## Conclusions

This study demonstrates that younger patients and those with DCIS undergoing BCS for breast cancer have a higher rate of positive margins. For such patients at higher risk of positive margins, excision of cavity shave margins and intraoperative inking may be employed as strategies to lower positive margin rates and guide re-excisions, respectively. Preoperative review of breast imaging, core biopsies, and counseling of patients about the likelihood of positive margins is important to reduce the need for a second surgery.
